# Concomitant Septate Hymen and Bicornuate Uterus: Emphasizing the Need for Thorough Internal Genital Tract Evaluation

**DOI:** 10.7759/cureus.109664

**Published:** 2026-05-26

**Authors:** Lisa Brinkman, Wendela Kolkman, Miriam Hanstede, Aukje van Tilborg

**Affiliations:** 1 Obstetrics and Gynaecology, Leiden University Medical Center, Leiden, NLD; 2 Obstetrics and Gynaecology, Haga Hospital, The Hague, NLD; 3 Obstetrics and Gynaecology, Spaarne Gasthuis, Haarlem, NLD; 4 Radiology, Haga Hospital, The Hague, NLD

**Keywords:** concomitent genital tract anomalies, female genital tract, hymenal anomalies, hymenectomy, pelvic magnetic resonance imaging, reproductive and obstetric outcomes, transvaginal ultrasound, uterine anomalies

## Abstract

Congenital anomalies of the hymen are uncommon, and their co-occurrence with congenital uterine anomalies is considered even rarer. This case report describes a patient with a septate hymen combined with a bicornuate uterus. A teenage girl presented to the emergency department with a retained intravaginal tampon. Local examination revealed a septate hymen, and hymenectomy was performed. Over the following years, multiple transabdominal pelvic ultrasounds were performed because of persistent functional hypothalamic amenorrhea, revealing no underlying abnormalities of the internal genital tract. However, several years later, a transvaginal ultrasound performed after the insertion of an intrauterine device suggested the presence of a uterine anomaly. The diagnosis of a bicornuate uterus was confirmed by magnetic resonance imaging and hysteroscopy. Thus, this clinical case highlights the necessity of a thorough internal genital tract evaluation in females diagnosed with congenital hymenal anomalies.

## Introduction

Congenital anomalies of the female genital tract are structural malformations arising from disruptions in embryonic development, with an estimated incidence of 4-7% in the general female population [1,2]. Increasing evidence regarding the negative effects of female genital tract anomalies on reproduction and psychosocial well-being highlights the need for early identification and management [1-3]. Although these insights are well recognized, the current literature still indicates an incomplete understanding of the embryological structures and processes involved in the physiological development of the female genital tract, as well as the disturbances that result in congenital anomalies [3]. In addition, the mechanisms underlying concomitant congenital anomalies remain unexplored in the available literature. To date, this gap in understanding has limited the development of scientifically well-founded clinical assessment strategies following the diagnosis of a female genital tract anomaly, particularly involving the hymen. Therefore, this case report aims to provide insight into the available evidence on the pathogenesis of congenital female genital tract anomalies and to illustrate how this case highlights potential overlapping developmental mechanisms that result in concomitant hymenal and uterine anomalies.

According to the current literature, the development of the female genital tract is a complex process that begins at approximately the fifth week of gestation and continues into the second trimester [4]. The Müllerian ducts, in contrast to the Wolffian ducts in males, and the urogenital sinus play pivotal roles in the formation of the female internal reproductive system [4,5]. The Müllerian ducts develop along the anterolateral surface of the urogenital ridge, which itself originates from the intermediate mesoderm [5]. In contrast, the urogenital sinus arises from the cloaca, an endoderm-lined cavity [5]. A precise sequence of events involving both structures, including cellular differentiation, migration, fusion, and canalization, is required to form a complete female genital tract [3]. Disturbances in these processes can result in congenital anomalies of the uterus, cervix, fallopian tubes, vagina, and hymen. Notably, the ovaries, which originate from the genital ridge, are generally not affected [3,6].

Physiologically, the hymen is a thin piece of mucosal tissue that surrounds or partially covers the vaginal introitus [7]. Completely occluding hymens are estimated to occur in 0.05-0.1% of female neonates, whereas subocclusive hymenal variants are observed in 0.7% of females [7]. To date, associations between hymenal and other congenital anomalies have been minimally studied.

Anatomically, the uterus is a hollow, inverted pear-shaped organ [1]. Congenital uterine anomalies are relatively common, with a prevalence of 5.5% in a large, unselected population [2]. Congenital uterine anomalies also vary in severity, ranging from an arcuate uterus to complete aplasia [3]. Notably, uterine anomalies are associated with congenital cervical and vaginal anomalies, likely because of their shared embryologic origin and developmental processes [1,3]. In addition, uterine anomalies co-occur with renal tract abnormalities, specifically unilateral renal agenesis, with an estimated prevalence of 11.2% [8]. Accordingly, evaluation of the internal genital and renal tracts is generally considered an important step following the diagnosis of a uterine anomaly.

## Case presentation

A teenage girl presented to the ED with a retained intravaginal tampon after she forgot to pull out the string before insertion. Attempts to retrieve the tampon using a speculum were painful and unsuccessful. After an hour, the tampon was eventually removed, revealing that it was caught on a fibrous strand. This led to the diagnosis of a septate hymen, and a few months later, a hymenectomy was performed. No further evaluation of the internal genital tract was conducted at that time.

In subsequent years, the patient suffered from functional hypothalamic amenorrhea. Physical examination revealed Tanner stage IV, along with normal-appearing labia majora and minora and a normal vaginal introitus. Repeated transabdominal pelvic ultrasounds showed no abnormalities of the internal genital tract. A pituitary MRI scan, performed to evaluate potential hypothalamic-pituitary abnormalities as a cause of her amenorrhea, showed no abnormalities. After three years, her menstrual cycle resumed spontaneously following a reduction in her exercise intensity.

Several years later, during the placement of a copper intrauterine device, a discrepancy in uterine length measurements before and after insertion raised concerns about potential uterine perforation. The patient was referred to a gynecologist for an emergency transvaginal ultrasound. The ultrasound showed no signs of uterine perforation. However, the intrauterine device was only visible on the left side of the uterus, raising suspicion of a uterine anomaly. Subsequent MRI of the pelvis confirmed the presence of a uterine anomaly with no associated cervicovaginal or renal tract abnormalities. Hysteroscopic findings were concordant with those on pelvic MRI.

Subsequently, anatomical findings from both imaging and clinical assessments were used to classify the observed abnormalities in this patient. Congenital anomalies of the female genital tract are classified according to the European Society of Human Reproduction and Embryology (ESHRE)/European Society for Gynecological Endoscopy (ESGE) classification system, which provides a standardized framework for describing the observed uterine (U), cervical (C), and vaginal (V) anatomy [1]. The uterine class reflects fundal contour and cavity morphology (U0-U6), the cervical class defines normal cervical anatomy, a septate or doubled cervix, or cervical aplasia (C0-C4), and the vaginal class describes normal vaginal anatomy, longitudinal or transverse septa, or vaginal aplasia (V0-V4) [1]. Based on all findings, this patient was diagnosed with a bicornuate uterus with a uterine septum extending to the uterocervical canal (U3c), with no associated cervical (C0) or vaginal (V0) anomalies, corresponding to U3cC0V0 according to the ESHRE/ESGE classification (Figures 1-2) [1].

**Figure 1:**
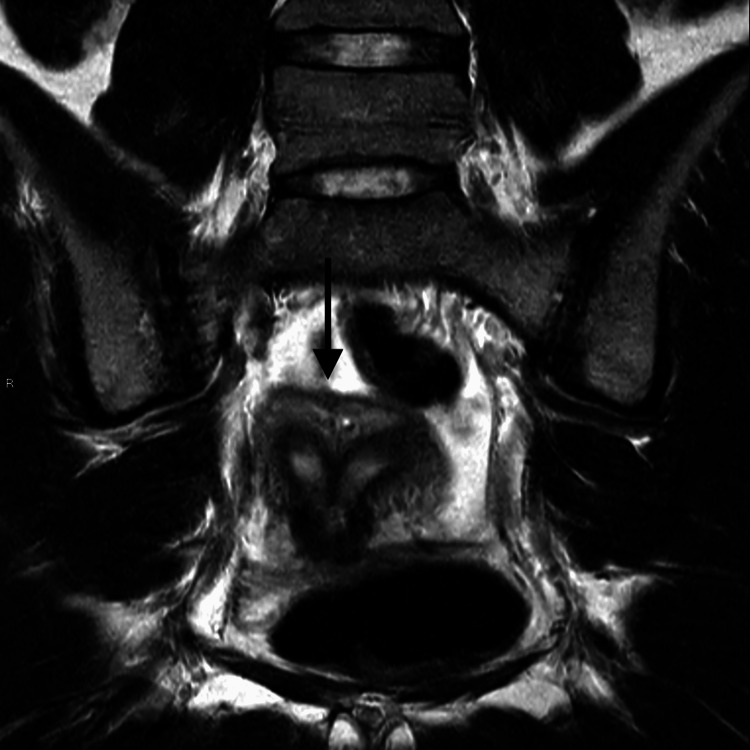
Coronal pelvic MRI demonstrating a bicornuate uterus. Coronal pelvic MRI demonstrating a bicornuate uterus with a septum extending to the uterocervical canal, classified as U3cC0V0 according to the ESHRE/ESGE classification system [1]. ESHRE/ESGE: European Society of Human Reproduction and Embryology/European Society for Gynecological Endoscopy classification system.

**Figure 2:**
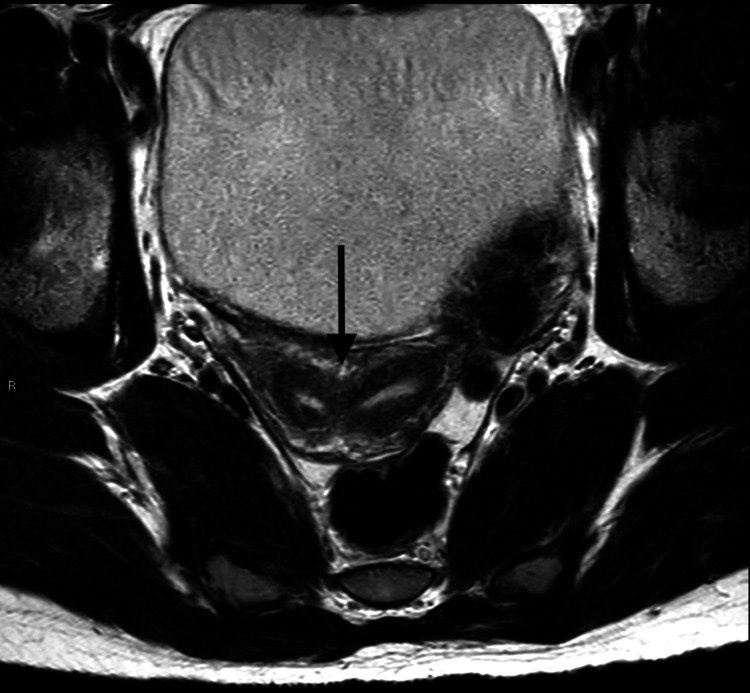
Axial pelvic MRI demonstrating a bicornuate uterus. Axial pelvic magnetic resonance imaging demonstrating a bicornuate uterus with a septum extending to the uterocervical canal, classified as U3cC0V0 according to the ESHRE/ESGE classification system [1]. ESHRE/ESGE: European Society of Human Reproduction and Embryology/European Society for Gynecological Endoscopy.

## Discussion

While cases of isolated congenital anomalies of the female genital tract are relatively well documented, the literature on concomitant hymenal and uterine anomalies remains limited. Furthermore, the etiology of hymenal anomalies is largely unknown. This is due to the incomplete understanding of the physiological development of the hymen, as even its embryological origin remains uncertain [9]. The embryonic hymen is thought to form at the junction of the urogenital sinus and the lumen of the canalizing vagina [3,9]. Some studies suggest that the hymen likely arises from the urogenital sinus in response to contact with the Müllerian ducts, forming from central cells of the Müllerian tubercle that fail to disintegrate [9,10]. According to other theories, the hymen arises solely from the urogenital sinus, specifically via passive invagination of the urogenital sinus wall [11]. The hymen is initially imperforate during early embryonic development and typically ruptures prenatally through degeneration of the central epithelial cells [7]. The physiological mechanism of hymenal rupture is likely anterior, since microperforate hymens consistently exhibit anterior openings [12]. The exact timing of rupture is unclear, but it is likely before 23 weeks of gestation, as a large observational study of neonates born between 24 and 42 weeks showed no variation in hymenal morphology related to gestational age or birth weight [13]. Unsuccessful canalization of the vaginal plate due to failure of degeneration or excessive cellular proliferation and coalescence of the hymenal epithelial cells may underlie hymenal anomalies [14].

Compared with the hymen, the embryonic development of the uterus is well understood. The uterus develops after the two Müllerian ducts have migrated, fused, and canalized within the first five months of gestation [3]. Developmental defects such as Müllerian agenesis, failure of fusion, and failure of canalization of the Müllerian ducts result in congenital uterine anomalies [3]. Müllerian agenesis, also referred to as Mayer-Rokitansky-Küster-Hauser syndrome, is characterized by the absence of the uterus and the upper portion of the vagina [6]. A unicornuate uterus results from the failure of one Müllerian duct to develop or reach its proper position during early embryonic development [3]. Failure of vertical fusion of the Müllerian ducts and urogenital sinus can lead to a transverse vaginal septum or absence of the cervix [3]. Failure of lateral fusion of the Müllerian ducts results in uterine anomalies such as bicornuate or didelphys uteri [3]. Following fusion, incomplete resorption of the adjacent walls of the Müllerian ducts results in a persistent division of the uterine cavity, giving rise to a subseptate or, in some cases, complete septate uterus [3]. The etiology of uterine anomalies remains largely unknown but is thought to be multifactorial, comprising genetic factors together with environmental factors [3].

Consistent with this case report, several other case reports and reviews describe a clinical association between hymenal and uterine anomalies. These studies document multiple patients with various forms of hymenal anomalies combined with different types of uterine anomalies [15-18]. Interestingly, a long-term follow-up of females previously diagnosed with an imperforate hymen showed concomitant uterine anomalies, including bicornuate and didelphys uteri, in 13% of cases [15]. Another study of young females with congenital vaginal agenesis, most with concurrent uterine agenesis, reported hymenal anomalies in 49% of the females, a markedly higher rate than in the general population [16]. The observed hymenal anomalies included septate, cribriform, microperforate, and absent hymens [16]. The presence of Müllerian agenesis in combination with hymenal anomalies, especially absent hymens, suggests that Müllerian ducts may also play a role in the embryonic development of the hymen [16-18].

To the best of our knowledge, no validated overlapping factors or processes contributing to the co-development of hymenal and uterine anomalies have been described in the available literature. Moreover, the uncertainty regarding the structural origin of the hymen complicates understanding from an embryologic perspective. As illustrated, some researchers propose that the hymen originates solely from the urogenital sinus, while others suggest that it also involves the lateral parts of the Müllerian ducts [9-11]. In the latter case, since the uterus originates from the Müllerian ducts, structural or developmental abnormalities of these ducts may explain the co-occurrence of hymenal and uterine anomalies.

Although no validated mechanism has been established, the observed clinical association suggests a link in the embryonic development of both structures. Accordingly, the diagnosis of a hymenal anomaly may increase the likelihood of identifying a concurrent uterine anomaly. Hymenal anomalies are often detected during puberty because of their interference with menstruation, coitus, or fertility [17]. In contrast, uterine anomalies are frequently asymptomatic, particularly before the first gestation, and are incidentally detected during this period [3]. However, since uterine anomalies are associated with obstetric complications, such as an increased risk of preterm delivery and fetal malpresentation, their identification before gestation is crucial for enabling timely follow-up and interventions [2,3]. Consequently, the diagnosis of a hymenal anomaly should prompt further investigation for the presence of other internal genital tract abnormalities, particularly of the uterus.

Currently, protocols following the diagnosis of hymenal anomalies vary widely. While some gynecologists limit their treatment plan to hymenectomy, others recommend additional imaging, such as transabdominal pelvic ultrasound, pelvic MRI, or hysteroscopy. Widely established guidelines on hymenal anomalies do not mention the clinical association with uterine anomalies, nor do they explicitly recommend additional examination of the internal genital tract [19,20]. Moreover, the American College of Obstetricians and Gynecologists states that, following the diagnosis of an imperforate hymen with typical findings, further imaging or investigation of the internal genital tract is not necessary [20]. However, given the reported clinical association, a thorough internal genital evaluation is recommended for females diagnosed with hymenal anomalies.

## Conclusions

The etiology of congenital female genital tract anomalies, particularly concomitant anomalies, remains largely undetermined. This case report, combined with the current literature, demonstrates a clinical association between congenital hymenal and uterine anomalies. Therefore, identification of a hymenal anomaly should prompt a thorough evaluation of the internal genital tract, especially the uterus, preferably through 3D transvaginal ultrasound or pelvic MRI. This is of particular importance given that early and accurate diagnosis of potential coexisting uterine anomalies is crucial for optimizing reproductive and obstetric outcomes.
